# Hydration Meets Regulation: Insights into Bicarbonate Mineral Water and Acid–Base Balance

**DOI:** 10.3390/nu17142291

**Published:** 2025-07-10

**Authors:** Katharina Mansouri, Thierry Hanh, Andreas Hahn

**Affiliations:** 1 Institute of Food and One Health, Leibniz University Hanover, 30159 Hanover, Germany; mansouri@foh.uni-hannover.de; 2 Independent Researcher, 75016 Paris, France; thierry.hanh@gmail.com

**Keywords:** mineral water, bicarbonate, acid–base balance, urine, net acid excretion, blood gas, kidney stones, bones

## Abstract

Acid–base balance is critical to human health and can be significantly influenced by dietary choices. The Western diet, characterized by high meat and cheese consumption, induces excess acidity, highlighting the need for strategies to mitigate this. Recent studies have focused on bicarbonate-rich mineral water as a viable solution. In this context, the present narrative review synthesizes the findings from recent scientific studies on bicarbonate-rich mineral water, specifically those with bicarbonate levels over 1300 mg/L and medium or low PRAL values. This water has been shown to exert beneficial effects on both urinary and blood parameters. The key effects include an increase in the urine pH and a profound reduction in net acid excretion as a sign for a reduced acid load. Additionally, bicarbonate mineral water has been shown to decrease the excretion of nephrolithiasis-related constituents, including calcium and oxalates, as well as inhibitory substances such as magnesium and citrates. In blood, bicarbonate-rich water has been demonstrated to stabilize pH and increase bicarbonate levels, thereby enhancing systemic buffering capacity. Clinically, these changes have been associated with a lowered risk of calcium oxalate stone formation and improved kidney health. Furthermore, bicarbonate-rich water has been shown to support bone health by reducing bone resorption markers. Consequently, the integration of bicarbonate-rich mineral water into the diet has the potential to enhance urinary and blood parameters, mitigate the risk of kidney stones, and strengthen skeletal integrity, thereby serving as a promising strategy for health promotion and disease prevention. While promising, these findings underscore the need for further research to establish long-term recommendations. Future interventional studies should be designed with rigorous randomization, larger sample sizes, cross-over methodologies, and comprehensive dietary assessments to address the methodological limitations of previous research.

## 1. Introduction

The maintenance of the acid–base balance in the body represents a fundamental aspect of physiological homeostasis, essential for life. Blood pH, typically ranging between 7.35 and 7.45 (arterial) [1], is carefully regulated to maintain cellular functions, enzymatic activity, and overall metabolic integrity [2]. Any deviation from this narrow pH range results in significant health consequences [3], manifesting as either acidosis or alkalosis. The lungs and kidneys serve as the primary organs that are crucial for the regulation of acid–base homeostasis [1]. Specifically, volatile acids are excreted via the lungs as carbon dioxide, whereas non-volatile acids must be excreted via the kidneys [4].

Diet composition significantly affects acid–base balance [1]. Different dietary components can either contribute to endogenous acid production or provide alkaline precursors [5]. Food rich in protein, particularly those with sulfur-containing amino acids and phosphorus, such as meat, cheese and eggs, tend to increase acid load upon metabolic processing [6]. By contrast, fruits and vegetables, particularly those rich in potassium and magnesium, provide alkaline precursors, and metabolization promotes a more alkaline environment [1,6,7]. Although grain products contain an average amount of sulfur-containing proteins [4], they still contribute to an increase in acid load. Therefore, grains should be considered as acidifying foods because of their comparatively high intake. Due to a high protein consumption and the lack of fruits and vegetables, the modern Western diet is considered to cause an excess of acid equivalents [6,7,8]. Consequently, such dietary patterns are suggested to cause imbalances in the acid–base balance in the long-term [4] resulting in a low-grade metabolic acidosis.

This narrative review aims to give a short overview of the acid–base balance and the association between high acid load and health consequences in general. However, the main part deals with the effects of bicarbonate-rich mineral water consumption on acid–base balance, as this water deliver higher amounts of bicarbonate potentially modulating the parameters of acid–base homeostasis.

## 2. Literature Search Strategy

To identify the relevant literature for this narrative review, a comprehensive search of the electronic database PubMed was conducted. Reference lists of selected articles were also screened to identify additional relevant publications. The focus of this review is on the literature published in English, with a few exceptions. Therefore, the search was limited to articles published in English. However, relevant references in French and Italian were identified in the reference list of the selected publications. Therefore, these studies were also included. The following keywords and keyword combinations were employed: “mineral water”, “water”, “bicarbonate”, “acid-base”, “blood gas”, and “pH”. In addition, the Boolean operators “AND” and “OR” should be used. Only full-text versions of the literature that reported on the relationship between bicarbonate-rich mineral water and acid–base balance were included. This encompasses studies on classical acid–base parameters in urine (acid excretion) and blood (blood gas) as well as related studies on kidney stones and bones.

## 3. Intake of Acid Precursors and Acid Excretion

The acidizing or alkalizing potential of foods and beverages is usually estimated using equations developed for this purpose. In general, these estimations show a good correlation to the directly measured renal net acid excretion [4,9]. However, their accuracy can be questioned at the individual level, due to limitations in the precise measurement of dietary intake, the individual absorption of nutrients in the gastrointestinal tract, and the nutrient composition of foods [4]. Nevertheless, three equations or measurements are frequently used in nutritional studies to assess the effect of foods and beverages on the acid–base balance: the Potential Renal Acid Load (PRAL) score, net endogenous acid production (NEAP), and net acid excretion (NAE). These parameters offer valuable insights into the complex interplay of dietary intake, endogenous metabolism, and renal function in maintaining acid–base balance.

Foods and beverages usually contain acid precursors, with fruits and vegetables additionally containing base precursors [10]. The **PRAL** calculation of foods and beverages is a commonly used method developed to estimate the acid or base forming potential of the foods [11], expressed in milliequivalents per 100 g (mEq/100 g). This value reflects the amount of acid excreted by the kidneys when 100 g of a food is consumed [1,12]. The PRAL values of different foods can vary between negative and positive values. Negative values indicate a more alkaline potential and positive values indicate a more acidic potential [1,13]. In general, the more negative the PRAL value, the less acid needs to be excreted via urine [12]. The formula for the calculation of PRAL was developed by Remer and Manz in the 1990s. It takes into account the average intestinal absorption rate of the various nutrients that indicate an acidifying or alkalizing effect of food. In addition to the amount of the predominant anions (phosphorus, protein, and chloride), the amount of the predominant cations (magnesium, calcium, potassium, and sodium) is integrated in the original formula (PRAL_R_) [11]. Although six minerals were initially considered, it has been demonstrated that the PRAL estimation using only four minerals (phosphorus, magnesium, calcium, and potassium) allows for a simpler assessment of dietary acidity. Both methods showed a reasonable agreement with the measured NAE. In this formula chloride and sodium are omitted, due to large inaccuracies in estimating sodium and chloride intake via processed foods in food tables [14]. A second, slightly modified equation was developed by Sebastian et al. (PRAL_S_) [15], taking into account the sulfate content of the diet instead of the protein content.

However, the PRAL value does not completely reflect the renal net acid excretion, as it excludes endogenously synthesized organic acids (OAs) in the equation. In order to enhance the precision of quantifying the total acid excreted in urine, the **NEAP** (net endogenous acid production) estimation was developed by Remer and Manz [11] and modified by Lemann [16] and Sebastian et al. [15]. However, all equations include estimated OAs: NEAP = PRAL + OA. The estimated organic acids thereby reflect an anthropometry-based estimation using individual body surface (OA_anthro_) [11] or the dietary intake of the main anions and cations (OA_diet_) [16] for the calculation (Table 1).

In addition, a simplified algorithm has been developed by Frassetto et al. [17], focusing on the two primary determinants of endogenous acid production, protein intake for acid production and potassium intake for base production, as they are the major variable components and independent predictors for NEAP. While protein is an indicator for the rate of sulfuric acid production, the potassium salts of organic acids are an indicator of bicarbonate generation upon metabolism [18]. There are two equations: NEAP_F_ = protein—potassium or protein/potassium. Both equations from Frassetto et al. offer the possibility to estimate NEAP under conditions of restricted information on dietary nutrient and mineral content. Nonetheless, an apparent discrepancy exists among the calculation models in terms of their validity in relation to the measured NAE. The calculation of NEAP using the equations by Remer (NEAP_R_) and Lemann (NEAP_L_) showed a good agreement with NAE, although both equations were imprecise. However, the equations by Sebastian (NEAP_S_) and Frassetto (NEAP_F_) did not provide reasonable correlations [19].

In general, the NEAP values vary widely between individuals, typically ranging from 20 mEq to 120 mEq per day [20]. However, contemporary Western diets usually lead to a total acid load of about 50–100 mEq/d [11,13,21,22]. By contrast, the ingestion of a vegan diet has been shown to result in a reduced acid excretion, as demonstrated by a lower mean NEAP value of −0.72 ± 29.8 mEq/day [23].

**Table 1:** Equations for the estimation of net endogenous acid production (NEAP).

Name of the Equation	Equation/Formula	Reference
**NEAP_R_** (mEq/d)	PRAL_R_ (mEq/d) * + OA_anthro_ (mEq/d)	[14]
OA_antho_ (mEq/d)	BSA ** (m^2^) × 41 (mEq/d)/1.73 (m^2^)	[11]
**NEAP_L_** (mEq/d) OA_diet_ (mEq/d)	PRAL_R_ (mEq/d) * + OA_diet_ (mEq/d) 32.9 + (0.15 × [{potassium} + {calcium × 2} + {magnesium × 2} − {phosphorus × 1.8}]) (all in mmol/d)	[16]
**NEAP_S_** (mEq/d) OA_diet_ (mEq/d)	PRAL_S_ (mEq/d) * + OA_diet_ (mEq/d) 32.9 + (0.15 × [{potassium} + {calcium × 2} + {magnesium × 2} − {phosphorus × 1.8}]) (all in mmol/d)	[15]
**NEAP_F_** (mEq/d)	Equation (1): [54.4 × protein (g/d)/potassium (mEq/d)] − 10.2 Equation (2): [0.91 × protein (g/d)] − [0.57 × potassium (mEq/d)] + 21	[17]

* using the simplified formula of potential renal acid load from Remer et al., omitting Cl and Na [14]; OA = endogenously produced organic acids (derived from anthropometric measurements or diet); ** BSA = individual body surface area, for example calculated by the formula of Du Bois and Du Bois [24]: BSA = 0.007184 × height (cm)^0.725^ × weight (kg)^0.425.^

In a “steady state”, NEAP is equivalent to renal net acid excretion (**NAE**) [2,6,17], encompassing both dietary acid load and endogenous acid production. Therefore, NEAP is reflected in the net result of the acid–base regulatory processes. Unlike PRAL and NEAP, which estimate acid production resulting from food intake, NAE can be measured directly in 24-h urine samples to quantify the amount of acid excreted via urine. Its determination is based on the measurement of titratable acids, ammonium, and bicarbonate in the urine: NAE = TA + NH_4_^+^ − HCO_3−_ [17].

In general, the renal acid excretion capacity surpasses the usual acid load derived from dietary intake. Consequently, significant diet-induced disturbances of the acid–base balance in the blood are uncommon in healthy individuals [10]. Excess acids are excreted via urine, which is reflected by a decrease in the urine pH [2]. Nonetheless, there are physiological limits; a decrease in the pH value to below 4.5 results in a reduction of H^+^ excretion due to impaired protein function in the proximal tubule. Furthermore, a urinary pH below 5.4 indicates a maximally stimulated acid excretion [2], which is associated with serious metabolic consequences [25]. Unlike healthy individuals, individuals with impaired renal function may experience clinically evident metabolic acidosis even with a lower dietary acid load [2,25]. This particularly affects individuals with chronic kidney disease and the elderly, whose ability to excrete acids decreases with increasing age [10].

Nevertheless, prolonged excessive intake of acidic precursors, as commonly observed in the typical Western diet [26], can result in chronic depletion of the body’s buffering systems. Maintaining a constant systemic pH to sustain essential biochemical processes can lead to alterations in the acid–base balance, even among healthy people. If the daily dietary acid load exceeds 1 mmol H^+^ per kilogram of body weight, the risk of a positive acid balance increases [16]. This physiological state is known by various terms, including “low-grade metabolic acidosis” [12], “chronic sub-clinical systemic metabolic acidosis (CSSMA)” [27], “subclinical acidosis” [28], “preclinical acidosis” [29], or “eubicarbonatemic acidosis” [30]. At this stage, there is a noticeable shift towards acidity, even though the pH of the blood is still within the normal range. Additionally, this condition is often accompanied by a slight decrease in the systemic bicarbonate pool and a notable increase in renal acid excretion [12]. If this condition persists over a long period of time, it may contribute to the development and aggravation of several diseases [1,12,27]. Small adaptations occur throughout the body; all aimed at eliminating excess acid equivalents. However, these adaptations do not occur without consequences for the organism in the long term [2].

## 4. Associations Between Dietary Acid Load and Metabolic Alterations or Diseases

**Figure 1:**
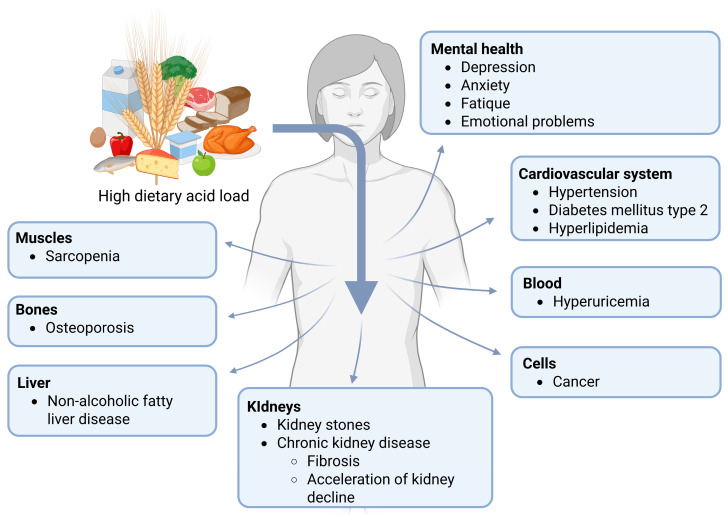
Discussed consequences of a high dietary acid load.

Numerous epidemiological studies have demonstrated an association between high dietary acid load and the occurrence of various metabolic alterations and diseases. On the one hand, increased cortisol levels are shown in individuals with high dietary acid load [31,32]. In addition, serum uric acid levels are increased [33,34] and the risk of hyperuricemia is elevated [35]. On the other hand, increases in the urinary excretion of calcium [36] and ammonium [37] are demonstrated, while citrate excretion decreases [38,39], accompanied by a decrease in the urinary pH [40,41]. Furthermore, the synthesis of endothelin-1, angiotensin II, and aldosterone in the kidney is stimulated, thereby activating several pro-fibrotic factors [1]. These alterations are accompanied by systemic changes affecting the kidneys, bones, muscles, liver, and the endocrine system (Figure 1).

## 5. Mineral Water

Mineral water is a calorie-free beverage that can contribute significantly to hydration. Beyond its role in maintaining fluid balance, mineral water provides essential minerals, such as calcium and magnesium. Consequently, its consumption offers a range of potential health benefits that extend beyond mere caloric considerations. Several intervention studies have shown the beneficial effects of mineral water consumption in supporting the treatment of kidney stones [42], osteoporosis [43], metabolic syndrome [44], cardiovascular diseases [45], and digestive disorders [46]. In addition, mineral water consumption has been associated with improved exercise performance and rehydration [47,48]. These benefits are attributed to several underlying mechanisms, including its effect on the acid–base balance [43,44], its stimulation of intestinal activity through osmotically active compounds [46,49], and its contribution to the supply status of various minerals [50]. However, it is important to note that the health benefits of mineral water vary significantly depending on the amount of dissolved ions. Mineral water used to relieve digestive disorders, such as constipation, may not necessarily provide benefits in the prevention of kidney stones [51]. In addition, mineral water that is effective against calcium oxalate stones have proven to be counterproductive against magnesium ammonium phosphate and calcium phosphate stones [52]. Therefore, the specific composition of each mineral water explains its supportive or counterproductive effects in terms of treatment support and symptom alleviation

## 6. Bicarbonate-Rich Mineral Water

**Figure 2:**
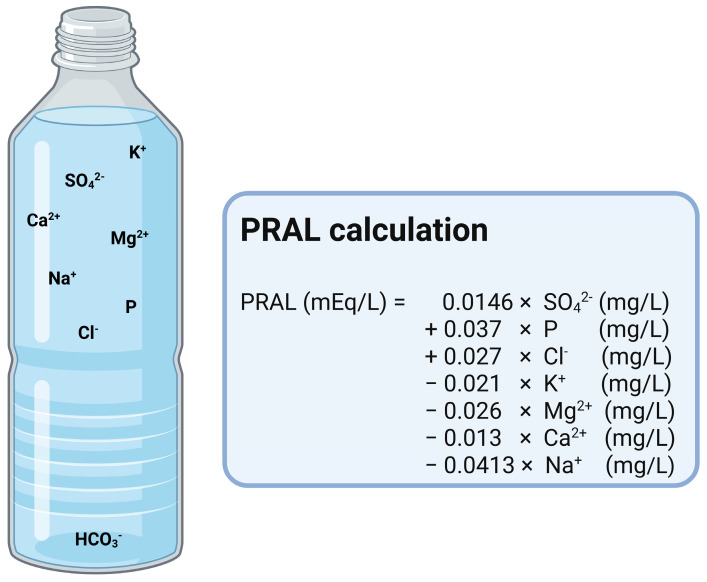
PRAL calculation for mineral water [56].

Most mineral water contains lower amounts of bicarbonate (50–100 mg/L). However, there is mineral water with much higher bicarbonate levels, reaching several grams per liter [53]. In a recent study examining the composition of several commercial mineral water brands available in 10 European countries, the mineral water with the lowest bicarbonate levels were sold in Italy and United Kingdom, while those with the highest bicarbonate levels were sold in France and Spain. Poland had the highest calculated average level of bicarbonate in the mineral water on the market [54,55], indicating several mineral water brands with a high bicarbonate content in this European country. However, the mineral water with the highest bicarbonate level included in this study was sold in France, showing a bicarbonate content of 4368 mg/L [55]. In this context, mineral water with a high concentration of bicarbonate contains a minimum amount of at least 600 milligrams per liter. However, studies on its effects regarding health issues have been conducted utilizing mineral water with a bicarbonate content of approximately 1000 mg/L and above. The relationships between the components of mineral water differ fundamentally between alkalizing and acidifying water [51]. Bicarbonate-rich mineral water is known to have an alkalizing effect on human urine [43], similar to medication [52]. In order to estimate the alkalinizing or acidifying potential of mineral water (PRAL), modifications have been made to the original calculation model by Remer and colleagues in the literature [11]. Wynn et al. [56] substituted the amount of protein by the sulfate content of the mineral water. The original equation for the PRAL calculation used the protein content of food to estimate the acidifying effect of sulfate. In food, sulfate originates from cysteine and methionine, which are ingested along with dietary protein. However, in mineral water where sulfate is in solution, the molecular weight and absorption rate of the sulfate must be considered, not those of the amino acids from the dietary proteins [57]. This requires the substitution of protein and sulfate in the calculation [56] (Figure 2).

Given that bicarbonate and sulfate typically do not coexist in the same geological layer, bicarbonate-rich water typically contains minimal amounts of highly acidifying sulfate [51]. This explains the strong correlation between the PRAL value of a mineral water and its bicarbonate content [43]. In a study evaluating the relationship between dissolved ions in mineral water and its PRAL value, bicarbonate showed the strongest negative correlation with the calculated PRAL value [51]. This finding suggests that, despite the omission of bicarbonate content from the PRAL calculations, it may nevertheless exert a significant influence on the acid–base balance, in addition to the contributions of alkalizing and acidifying ions present in mineral water. It has been established that the substance is absorbed by the body in certain quantities, and subsequently excreted via the urine [53].

Moreover, alkalizing mineral water often contains high amounts of calcium. However, alkalizing water is not necessarily rich in calcium. Rather, some bicarbonate-rich water is also characterized by a high sodium content [42], which is shown in Table 2.

## 7. Bicarbonate-Rich Mineral Water and Human Health

### 7.1. Urinary Parameters

#### 7.1.1. Urine pH and Net Acid Excretion (NAE)

Several authors have stated that there is a beneficial effect of consuming bicarbonate-rich mineral water on the acid–base balance, both in terms of the urine [13,56,58] and the blood parameters [13,48,58,59].

Under normal physiological conditions, human urine is slightly acidic [52], a characteristic influenced by the intake of food and beverages. Nevertheless, there are conditions requiring a more alkaline pH to prevent unfavorable conditions or diseases, such as the occurrence of calcium oxalate and uric acid stones. Against this background, several studies have been conducted to evaluate the effects of bicarbonate supplements on urinary composition. Supplement intake has been demonstrated to lead to an increase in the urinary pH [60,61,62,63] and a reduction in renal net acid excretion [63,64,65,66,67,68,69]. However, the use of supplements containing bicarbonate is not quite common, whereas mineral water with a high content in bicarbonate can contribute to maintain the acid–base balance as well as to achieve hydration.

#### 7.1.2. Effects of Bicarbonate-Rich Mineral Water on Urine pH and NAE

**Figure 3:**
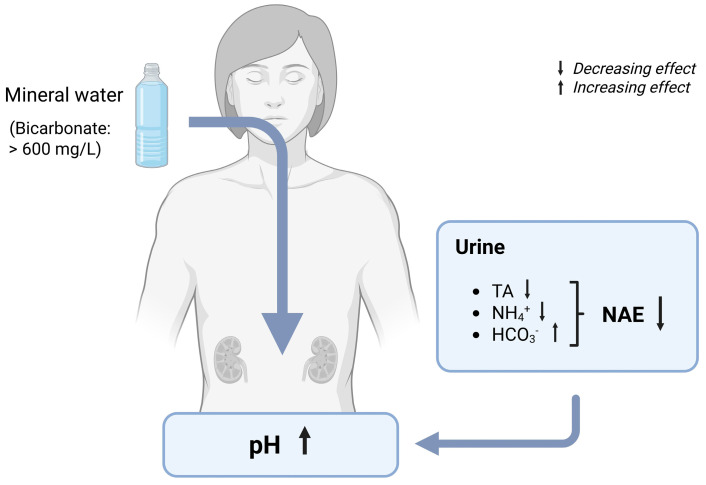
Effects of bicarbonate-rich mineral water on the urinary parameters. TA = titrable acids, NH_4_^+^ = ammonium, HCO_3_^−^ = bicarbonate, NAE = net acid excretion.

In recent decades, several studies have examined the impact of bicarbonate-rich mineral water on various aspects of urine composition (Table 3). These investigations have consistently reported favorable outcomes concerning the urinary pH and renal net acid excretion (NAE) (Figure 3). However, it should be noted that the majority of the studies only included a small number of participants (<50 subjects: [48,56,59,70,71,72,73,74,75,76,77,78,79,80], which limits the generalizability of the results. Moreover, only a very small part of the studies were conducted in a double-blind manner [59,70,77,79]. This might have affected the results, as the absence of blinding introduces a significant risk of bias, including the potential for falsification and exaggeration.

Bicarbonate-rich mineral water has been consistently demonstrated to have an alkalizing effect on urine. This effect has been demonstrated in numerous studies analyzing 24-h urine samples, as well as spontaneous or 2-h fasting urine [13,48,56,58,59,71,73,75,76,77,78,79,82,85,86]. Only two studies did not observe a significant change in the urinary pH following mineral water intake [72,74]. Remarkably, in both studies, the pH was measured in specimens differently from 24-h urine. Instead, measurements were carried out in morning fasting urine and spontaneous urine, potentially contributing to the absence of the observed changes in pH. An increase in the urinary pH has been observed following sub-chronic consumption of bicarbonate-rich mineral water, with intake ranging from 1000 mL to 2000 mL per day over periods spanning three days to twelve weeks. For example, one study demonstrated a rapid and sustained rise in the urinary pH throughout the day after mineral water intake. This observation was derived from the collection of urine on multiple occasions over the course of a 24-h cycle (with three-hour collections during the day and nine-hour collections at night) with the objective of evaluating the circadian rhythm [75]. This finding is supported by research showing that the consumption of bicarbonate-rich mineral water resulted in a significant increase in the urinary pH after just three days, whereas control water had no such effect. Similar outcomes were observed after a four-week consumption period in the same study. This indicates a rapid response with a long-lasting effect [58]. Furthermore, the alkalizing effect of such type of mineral water has been found to be comparable to that of potassium citrate supplementation [73].

There are two contributors to the effect of mineral water on the urinary pH. On the one hand, the amount of bicarbonate in the mineral water determines the level of effect on the urinary pH [52]. On the other hand, the alkalinity of the mineral water (PRAL value) also affects the urinary pH. Therefore, the interplay between bicarbonate content and mineralization seems to affect the urinary pH. To evaluate the possible differences, Wasserfurth et al. [13] conducted a study in which they examined the impact of three mineral water brands with varying bicarbonate content and the PRAL values on healthy, omnivorous adults. The urinary pH was affected by all three types of bicarbonate-rich mineral water. However, differences occurred. The water with low PRAL values led to significant pH increases regardless of the bicarbonate content (2451 mg/L and 1846 mg/L), whereas the water with a medium PRAL value produced only a marginally non-significant change. The authors concluded that PRAL may be more important than bicarbonate concentration in determining urinary alkalization.

In the same study, the consumption of different bicarbonate-rich mineral water brands resulted in a notable decrease in net acid excretion (NAE), attributed to a reduction in urinary titratable acids and ammonium. Interestingly, the advantageous impact on NAE was particularly pronounced among the groups consuming the bicarbonate-rich mineral water, characterized by a low potential renal acid load (PRAL) value. This is in line with a recent study, showing an even larger reduction in NAE after the consumption of a mineral water with a very low PRAL value and a very high bicarbonate content. As a result of daily mineral water intake over a period of 28 days, titratable acids were reduced to almost zero and ammonium excretion decreased by half. In addition, urinary bicarbonate excretion increased significantly [58]. There are additional studies demonstrating the beneficial effects on parts of the NAE equation, namely ammonium [75] and bicarbonate excretion [56]. In a study conducted with healthy male subjects, a significant decrease in urinary ammonium excretion was observed following a 5-day consumption of bicarbonate-rich mineral water while adhering to a standardized diet. The same effect was shown in a subsequent phase of this study, evaluating a 4-week mineral water consumption under the conditions of usual dietary and beverage consumption [75]. In an additional study, the consumption of bicarbonate-rich mineral water for a duration of four weeks resulted in a significant increase of urinary bicarbonate levels in healthy women [56]. However, both studies assessed only the components of the net acid excretion equation, focusing solely on either urinary ammonium or bicarbonate excretion. Hence, it is plausible that mineral water consumption may have influenced the unmeasured component, yet alterations in these parameters of the acid–base balance were not quantified.

The observed beneficial effects on NAE may potentially influence other urinary constituents, such as calcium excretion. A meta-analysis comprising five studies unveiled a statistically significant linear relationship between renal net acid excretion and urinary calcium excretion. Specifically, higher NAE levels were associated with elevated renal calcium excretion. For each 10-mEq increase in NAE, the urinary calcium increased by 0.3 mmol/d [88]. On the other side, a lower NAE value was accompanied by lower calcium excretion, which could be demonstrated for the intake of base supplements [36]. In line with these findings, the consumption of bicarbonate-rich mineral water has been demonstrated to reduce the urinary calcium excretion [70,76,87]. Nonetheless, mineral water with a calcium content between 170 and 548 mg/L has been shown to increase calcium excretion, presumably due to an overload in calcium intake [13,56,71,74,75,77,86]. Further information on this topic can be found in Section 7.2.2 on “stone risk”.

### 7.2. Mechanisms of Changed Urinary Composition by Bicarbonate-Rich Mineral Water

Besides the known effects of minerals on the acid–base balance, the consumption of bicarbonate via mineral water leads to an increase in the urine pH [42]. The increase in pH can be attributed to an increase in alkali reserve, which is caused by the intake of bicarbonate via mineral water [77]. Oral bicarbonate intake affects the acid–base equilibrium primarily due to its role as a buffer in the body [42]. When bicarbonate is administered, it increases the buffering capacity of the body; bicarbonate contributes to the primary human systemic buffer system—the plasma carbonic acid/bicarbonate buffer system— by direct intestinal absorption [2,89]. With an adequate buffer system in place, the protons generated during metabolism can be efficiently buffered and subsequently excreted via urine [2]. This also affects renal ammonia excretion. Ammoniagenesis potentially decreases as less acids need to be neutralized by the ammonia/ammonium buffer system in the kidneys. In addition, excess bicarbonate itself can be excreted directly via urine by upregulation of the switching cells (type B) in the collecting duct, contributing to urine alkalinization [90,91].

#### 7.2.1. Renal Stones

Among other things, dietary habits and the intake of certain medications significantly modulate the risk of kidney and urinary tract stones [92,93]. Most of them are related to acid–base balance. For example, the intake of magnesium and potassium is associated with a reduced risk of stone formation, while a higher intake of animal protein, sodium, and sucrose is associated with an increased risk of stone formation [38]. In addition, the daily consumption of mineral water has been shown to be associated with a reduced risk of kidney and urinary stones [93]. It is noteworthy that there are different types of urinary stones and the dietary causes of stone formation are as multifactorial as the different stone compositions. However, calcium oxalate (CaOx) stones are identified as the predominant causes of stones in over 80% of individuals with stone-related issues, often displaying a recurrent pattern [92]. Calcium phosphate stones (CaP), uric acid stones (UA), struvite stones, and cystine stones are less common in stone disease [94]. Since CaOx are the most common stones in the urinary tract, the focus of this section is on CaOx stones.

#### 7.2.2. Effects of Bicarbonate-Rich Mineral Water on Stone Risk

**Figure 4:**
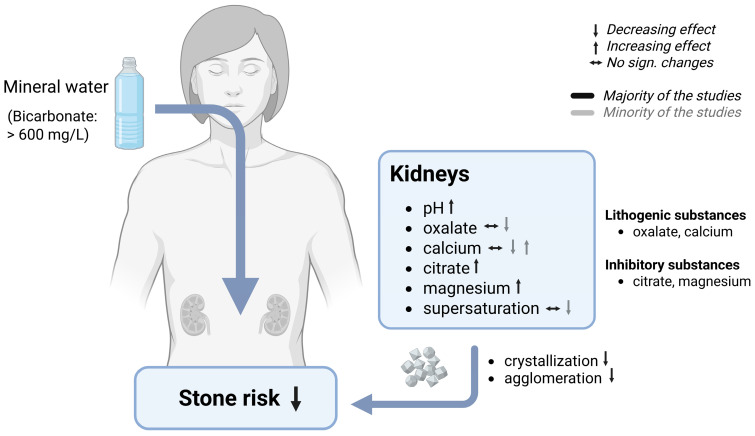
Effects of bicarbonate-rich mineral water on stone risk (CaOx/UA stones). Main effects are reported with black arrows. Grey arrows indicate effects in the minority of the studies. CaOx = calcium oxalate, UA = uric acid.

Among the various applications of bicarbonate-rich mineral water in the context of the acid–base balance, the prevention or recurrence of CaOx stone formation is by far the most extensively studied (Table 4) [71,72,74,75,77,81,86]. In this context, all studies on mineral water have focused on altering the composition of urine to establish a favorable balance between lithogenic and crystallization-inhibiting substances. In the past decades, the effect of bicarbonate-rich mineral water on stone risk was evaluated in several randomized controlled trials, showing mainly positive results (Figure 4). Most studies were conducted over a rather short or sub-chronical consumption period, ranging from three days to twelve weeks. In these studies, several urinary markers reflecting stone risk were tested, such as oxalates, calcium, citrates, and magnesium. Moreover, changes in the urinary pH were examined.

However, it is important to note that the majority of these studies had a limited sample size (<50 subjects: [56,70,71,72,73,74,75,76,77,95]), which reduces the extent to which their findings can be generalized. Moreover, only two studies were conducted in a double-blind manner [70,77]. This may have exerted an influence on the results, as the absence of blinding engenders a considerable risk of bias, including the potential for falsification and exaggeration.

The impact of bicarbonate-rich mineral water on raising the urinary pH is well-established and has been discussed in Section 7.1.1 on “urine pH”.

Besides urinary pH, one of the most important markers for the formation of CaOx stones is urinary **oxalate**. Its presence in increased concentrations has been associated with an elevated risk of stone formation [52]. The results of randomized controlled trials regarding the influence of bicarbonate-rich mineral water on urinary oxalate excretion are inconsistent. However, only a single study has demonstrated an increasing effect after a three-day consumption of 1500 mL mineral water in subjects with known urolithiasis [77], indicating a negative effect on stone risk. On the contrary, there are several studies showing a significant reduction in oxalate excretion after the daily consumption of 2000 mL bicarbonate-rich mineral water [71,72,73]. For instance, in one study conducted with healthy younger men, bicarbonate-rich mineral water demonstrated the same effectivity as a base supplement in reducing urinary oxalate concentrations under a standardized diet. While oxalate levels decreased by 10% following the consumption of K citrate, they were even lowered by 25% after the consumption of bicarbonate-rich mineral water. However, the authors claimed only a non-significant tendency towards a reduction under a usual diet [81]. Thus, dietary differences might be the main influencing factor regarding oxalate excretion. It is therefore not surprising that there are also studies showing no significant effect on the modulation of oxalate excretion in urine when food intake is not standardized [74,75,86]. It is worth mentioning that the effect of bicarbonate-rich mineral water on oxalate excretion could be influenced by both overall dietary intake and the calcium content of the ingested water. Furthermore, the timing of water consumption may be significant, particularly if the water is high in calcium. A high calcium intake during meals can alter oxalate excretion through the intestinal complexation of oxalates [86]. The higher the calcium intake during meals the lower the absorption rate of oxalates in the gut, thus a lesser amount of oxalates must be excreted via urine [96].

Besides urinary oxalates, urinary **calcium** excretion is also one of the important factors negatively affecting stone risk. Therefore, it could be assumed that a diet of mineral water low in calcium would be preferable for stone formers, as they do not involve an excessive intake of calcium that may need to be excreted via urine. However, there are studies indicating a decreased risk of stone formation associated with increased calcium intake [97,98]. This phenomenon may arise from the already mentioned effect of reduced oxalate absorption. Therefore, about half of the studies examining the impact on stone risk and bone health rely on the utilization of calcium-rich mineral water to augment dietary calcium intake. In some of these studies, the higher calcium intake resulted in an increase in urinary calcium excretion, possibly due to an excess in calcium intake [13,56,71,74,75,86]. However, the evaluation of group differences between bicarbonate-rich mineral water and the corresponding control water showed conflicting results. Three of the studies reported a higher calcium excretion in the bicarbonate group than the control group [71,74,75]. On the contrary, one study demonstrated lower levels after the consumption of bicarbonate-rich mineral water compared to a calcium- and sulfate-rich mineral water [55]. A further study underlined the influence of the calcium content of the studied mineral water brands. Groups receiving mineral water with a high calcium content showed a significant increase in calcium excretion, while the group consuming calcium-poor mineral water showed no significant changes in calcium excretion [13]. Nevertheless, there are also studies that have demonstrated no significant different effects on urinary calcium excretion, despite the use of calcium-rich mineral water in the bicarbonate-group [72,74,86]. In subjects with idiopathic calcium nephrolithiasis, the studied mineral water led to a non-significant rise in urinary calcium excretion after a 20-day consumption period [72]. In addition, one study reported a significant time × group interaction, showing the opposite effects in both intervention groups. While there was a non-significant rise in calcium excretion in the bicarbonate-group, the control group showed a decrease in calcium excretion [74]. In addition to the use of calcium-rich mineral water, there are also studies investigating the effect of bicarbonate-rich mineral water that was simultaneously low in calcium [58,70,76,77,81,85,95]. Surprisingly, one of these studies reported a significant increase in urinary calcium excretion in the bicarbonate group [77]. By contrast, no significant effects were demonstrated, although a tendency towards a reduction occurred [81]. In addition, there are several studies that have reported a significant reduction in calcium excretion [58,70,76,85,95]. However, only three studies on calcium-poor mineral water have reported a significantly lower calcium excretion in the bicarbonate group compared to the control group [76,85,95]. One could speculate that the discrepancies observed in the low-calcium mineral water could be due to differences in the dietary calcium intake. However, only seven out of fifteen studies have provided precise information on the dietary calcium intake, which makes it difficult to assess the influence of dietary calcium. The dietary interventions implemented in these studies were characterized by the provision of a standardized diet, which generally encompassed balanced calcium (approximately 1000 mg/d) and protein intake (75 to 96 g/d). Only two studies were conducted wherein the subjects followed a low-calcium diet (400–600 mg/d). Notwithstanding the implementation of a standardized diet, the outcomes pertaining to renal calcium excretion exhibited a great variability across these studies. Two studies have demonstrated no significant group differences [72,73], while others have reported a significant higher [71,74,75] or a significant lower calcium excretion in the bicarbonate group [56,95]. Taking into account the whole calcium intake (diet and mineral water), did not clarify the differences. The higher total calcium intake in the bicarbonate groups compared to the respective control groups [71,72,73,74,75] did not automatically result in a higher calcium excretion. Moreover, under both high [56] and low [95] calcium intake, consumption of bicarbonate-rich mineral water resulted in significantly lower calcium excretion compared to the control group that consumed the same amount of calcium. Therefore, the underlying causes seem to be independent from calcium intake.

In addition, urinary citrate and magnesium excretion play a pivotal role in stone inhibition [52]. A daily consumption of 1250–2000 mL of bicarbonate-rich mineral water has been shown to increase urinary **citrate** excretion in healthy subjects and stone formers, regardless the duration of the study [71,72,73,74,75,77,86]. Some of these studies have demonstrated the superiority of bicarbonate-rich mineral water over the control [71,75,77], while others have reported no significant group differences [72,73,74]. In addition, one study showed an opposite reaction in the two intervention groups, with an increase in the bicarbonate group and a decrease in the group consuming a low mineralized mineral water. However, no *p*-values are reported for time effects within each intervention group. Nevertheless, the differences in change over time were not significant (*p* = 0.084) [86].

Regarding **magnesium** excretion, several studies using bicarbonate-rich mineral water have demonstrated a beneficial effect. Participants who consumed 1250–2000 mL of this type of water showed a significant increase in urinary magnesium [71,72,75,77,86]. Changes occurred both in individuals without health issues [75] and those with a history of urinary stones [71,72,75,86]. Moreover, most of the studies have reported significant group differences for the used mineral water with a higher urinary magnesium excretion in the bicarbonate group compared to the control group. Only one study demonstrated a significant rise in magnesium excretion, which was lower in the bicarbonate group than the control group [72]. On the contrary, a minority of studies show no significant changes in urinary magnesium excretion [73,74], although magnesium excretion tended to increase in one of them (*p* = 0.058) [74]. Interestingly, these studies were conducted with healthy subjects, suggesting differences between stone formers and healthy participants.

Stone risk is usually assessed by evaluating the supersaturation of various urinary constituents, mainly CaOx and CaP. Although bicarbonate-rich mineral water positively affects the levels of various urinary constituents, its effect on stone risk appears to be inconsistent in terms of relative supersaturation. Despite the increased calcium excretion observed in some studies, the risk of stone formation was not adversely affected. On the contrary, the risk of CaOx stone formation was shown to decrease with the consumption of both calcium-rich and calcium-poor mineral water. Thus, beneficial effects were demonstrated for stone risk indices, especially for relative supersaturation and the Tiselius index [71,73,75,77], both methods for assessing the risk of recurrent stones [99]. For instance, in a study where the participants followed a standardized diet, the relative supersaturation of CaOx (RS CaOx) decreased by 43% after the consumption of bicarbonate-rich mineral water. This reduction was higher than the reduction in the control phase, where the participants received an alkaline supplement (K citrate). The control phase showed only a decrease of 25%. Moreover, the relative supersaturation of uric acid (RS UA) decreased by 73% in both study groups [73]. However, there are also studies that have showed positive changes in urinary composition, indicating a lower risk, despite the lack of significant effects on supersaturation [72,74,86]. One explanation for the absence of risk reduction in these studies could be the lack of significant reduction in urinary oxalates [74,86].

### 7.3. Mechanisms of Stone Risk Reduction by Bicarbonate-Rich Mineral Water

As a non-caloric beverage, bicarbonate-rich mineral water is one of the recommended beverages for subjects suffering from urolithiasis [42,52]. Increasing urine volume through higher fluid intake stands as the primary principle of stone metaphylaxis, with the aim of achieving a diluting effect on the urinary substances [42,52,100,101]. It is essential to augment fluid consumption systematically across diurnal and nocturnal periods to optimize urinary tract flushing, thereby reducing the retention time of urine in the urinary tract. This makes crystallization more difficult over the whole course of the day [52]. Indeed, a meta-analysis has showed that raising the fluid intake to achieve a urinary output ≥2500 mL/d reduced stone recurrence rates in patients with a history of nephrolithiasis [102].

As already stated, urinary pH is also an important factor contributing to stone risk. It influences the solubility of lithogenic substances, hence influencing crystallization and agglomeration. While CaOx stones and uric stones need a more acidic pH for crystallization, CaP stones and struvite stones are formed in a more alkaline pH [52]. Therefore, it is crucial to analyze the composition of stones in subjects suffering from urolithiasis to recommend the suitable mineral water. Gundermann et al. [52] stated that a bicarbonate amount of at least 1500 mg/L is necessary to raise the urinary pH in order to prevent CaOx or UA stones. Depending on the concentration of bicarbonate in mineral water and the consumed amount of mineral water, urinary pH increases differently. In most of the mineral water studies an increase up to pH 5.9–6.9 was demonstrated. However, urinary pH can even increase beyond the neutral point after the consumption of bicarbonate-rich mineral water [58,103].

All the above-mentioned urinary constituents (oxalates, calcium, citrates, magnesium) can be positively influenced by the consumption of bicarbonate-rich mineral water contributing to a risk reduction. Urinary lithogenic substances (calcium, oxalates) are partly demonstrated to be lowered by the consumption of bicarbonate-rich mineral water, resulting in a decrease in relative supersaturation. As both substances contribute to stone formation, the relative importance of oxalate excretion compared to calcium excretion is crucial in determining the risk of stone formation. This is due to the fact that calcium is a weaker crystallization promoter compared to oxalates [52,72]. Moreover, the excretion of inhibitory substances (magnesium, citrate) has been shown to increase after the consumption of bicarbonate-rich mineral water. Regarding citrate excretion, a pH-dependent mechanism for citrate re-uptake has been identified, resulting in an increased citrate excretion under more alkaline tubular pH conditions [104]. This in turn affects the crystallization process [52]. The formation of complexes between citrate and calcium leads to a reduction in the concentration of CaOx crystals [105]. In addition, magnesium forms soluble complexes with oxalates, thereby reducing supersaturation of the oxalate [106].

In terms of calcium excretion there are several possible mechanisms postulated. On the one hand, the reduction in calcium excretion may be due to a direct renal mechanism [85], mainly an increased calcium reabsorption in the distal tubules [76]. On the other hand, it may be due to the reduction of bone resorption as a consequence of a more balanced acid–base equilibrium. For a detailed description of this postulated mechanism, please refer to Section 7.5.2 on “bicarbonate-rich mineral water and bone health”. In addition, it is also postulated that the reduced calcium excretion after the consumption of bicarbonate-rich mineral water may be due to a compensatory mechanism for a reduced calcium absorption in the gut. Due to the higher pH in the digestive tract after the consumption of bicarbonate-rich mineral water, a lower solubility of calcium may lead to a lower calcium absorption, thus there is no need to excrete excess calcium [76,85].

#### Blood Gas Parameters

**Figure 5:**
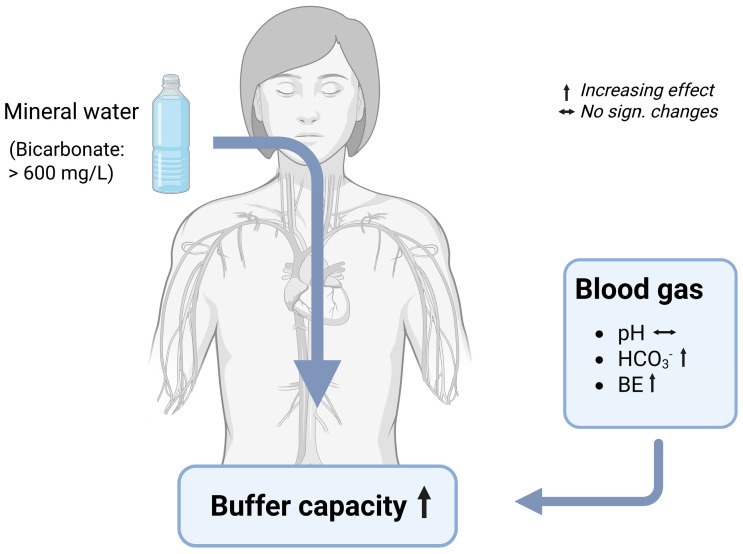
Effects of bicarbonate-rich mineral water on blood gas parameters. BE = Base excess, HCO_3_^−^ = bicarbonate.

Studies on the effects of mineral water with a high content in bicarbonate on blood gas parameters are rare. The effect on bicarbonate, pH, and base excess in capillary or venous blood have mainly been assessed in athletes, evaluating the potential to improve performance; whereas non-sport studies are scarce (Table 5, Figure 5).

In consideration of the sample size, it is important to note that only two studies have evaluated the effects in a larger study collective (>90 subjects: [13,58]), while the others were conducted with a maximum of 39 subjects [107]. Additionally, the absence of blinding in the larger ones [13,58] should be regarded as a limitation, which may have had an impact on the results.

The influence of bicarbonate-rich mineral water on the blood pH depends on the study conditions, as the results differ in some respects. On the one hand, there are beneficial effects on the **blood pH** after anaerobic exercise. Studies with recreationally active subjects were able to demonstrate a significantly higher blood pH immediately post exercise [59,107], 3 min and 5 min post exercise [59], and even 10 min post exercise [107] after a 7-day consumption of 1500 to 2000 mL bicarbonate-rich mineral water daily, in comparison to low mineralized mineral water. In another study, a single ingestion of 3000 mL bicarbonate-rich mineral water per day resulted in a significantly higher blood pH after anaerobic cycling and an isokinetic endurance test compared to the consumption of low mineralized mineral water [108]. This short-term consumption suggests a rapid effect in the context of anaerobic sports. In contrast to the findings observed after anaerobic exercise, results concerning resting pH demonstrate significant variation. A recent study demonstrated a significantly higher resting blood pH in highly trained athletes who consumed an alkalizing diet in combination with mineral water high in bicarbonate, in comparison to the control group who consumed mineral water low in bicarbonate [79]. However, on the other hand, the resting blood pH did not differ between the two intervention groups receiving bicarbonate-rich and control water for 21 days [48]. Consistent with the latter findings, studies outside the exercise-related context have shown no effects on the blood pH. One study conducted with 129 healthy subjects demonstrated significant group differences after the consumption of four different mineral water brands (1500–2000 mL/day) for four weeks. However, none of the mineral water showed significant increases in the blood pH [13]. In line with this, a recent study with 94 healthy subjects was not able to demonstrate significant changes in the blood pH after the consumption of 1500 to 2000 mL daily of bicarbonate-rich mineral water for four weeks, although the pH tended to increase over the first three days of mineral water consumption (*p* = 0.068) [58].

Besides blood pH, bicarbonate levels and base excess are valuable parameters to determine the acid–base balance in blood. Regarding **bicarbonate** levels in **blood gas** samples, there are conflicting results. In some of the studies, the consumption of bicarbonate-rich mineral water did not influence blood bicarbonate levels compared to low mineralized water [13,59,107], regardless of whether the parameter was measured in the context of exercise or not. However, there are also studies where the consumption of bicarbonate-rich mineral water led to significantly higher bicarbonate levels in the blood compared to the consumption of low mineralized mineral water [48,58,59,79,108]. The discrepancy may have occurred because of the different study designs. A study design with a higher bicarbonate intake via mineral water [48,79] and intake of mineral water during exercise [108] may be the reason for the positive findings.

The data on **base excess** (BE), a parameter that reflects the metabolic component of the acid–base balance by quantifying the amount of the excess or deficient base in the blood, is limited. Very few studies have evaluated the effect of bicarbonate-rich mineral water on the BE, with inconsistent results. An intake of about 3000 mg per day for 4 weeks did not change the base excess in healthy adults significantly, while a slightly higher bicarbonate intake (3677 mg/d) led to a significant increase in the base excess in the same study. However, there was no significant difference in the BE between the different study groups at the end of the study [13]. In addition, during anerobic cycling, the BE decreased equally in the bicarbonate group and the control group due to exercise [107]. On the contrary, a recent study was able to demonstrate a positive effect on the BE. In this study, conducted with healthy adults, the consumption of almost 7000 mg of bicarbonate per day from mineral water over a 4-week period led to a significant increase in the BE. The increase was observed after only 3 days of mineral water consumption and was maintained until the end of the study [58]. Similar results were obtained in a study on highly trained athletes. One hour after a 400 m run, the level of BE was found to be significantly higher in the group consuming an alkalizing diet in combination with mineral water high in bicarbonate, in comparison to the control group, who consumed mineral water low in bicarbonate in addition to an alkalizing diet [79]. Thus, the amount of bicarbonate ingested may contribute to positive or neutral effects in terms of the base excess.

### 7.4. Mechanisms of Changes in Blood Gas Parameters by Bicarbonate-Rich Mineral Water

Several mechanisms have been suggested to explain the beneficial effects of drinking bicarbonate-rich mineral water on blood gas parameters, in particular the increasing blood bicarbonate levels. When consumed, bicarbonate from mineral water interacts with hydrochloric acid in the stomach lumen, resulting in the formation of carbonic acid. This carbonic acid subsequently decomposes into water and carbon dioxide, with the latter being exhaled directly [109]. This mechanism likely explains the potential gastrointestinal side effects associated with the consumption of bicarbonate supplements, such as flatulence and belching [89]. Furthermore, it is hypothesized that some of the bicarbonate is absorbed into the bloodstream via stimulation of the basolateral Cl^−^/HCO_3_^−^ antiporter in the stomach, thereby increasing systemic bicarbonate levels [89]. At sufficiently high intake levels, bicarbonate also appears to pass through the stomach. This bicarbonate influx continues into the small intestine, where it is absorbed in the jejunum [110]. Once absorbed in the bloodstream bicarbonate acts as part of the bicarbonate–carbon dioxide buffer system, the physiologically most important buffer system in the human body [111].

#### 7.4.1. Bone Health

Diet and lifestyle factors are known to influence bone health, with several nutrients playing a key role in protecting human bones [8]. Among these nutrients, calcium is recognized as a bone-protective nutrient present in various foods and beverages [112,113,114]. Studies have shown that the bioavailability of calcium from mineral water is comparable to that from milk and dairy products [115,116]. Consequently, research using calcium-rich mineral water has been able to demonstrate a bone-protective effect in humans. However, there is evidence that this effect is not solely due to the calcium content; rather, the bicarbonate content of the mineral water also seems to play an important role [56,57]. As mentioned above, calcium-rich mineral water is either rich in bicarbonate or sulfate [56], resulting in either an alkalinizing or an acidifying effect on the body [43]. Therefore, the consumption of calcium- and bicarbonate-rich mineral water has the potential to counteract the acidogenic effects of an acidic diet, whereas a mineral water rich in calcium and sulfate can aggravate its adverse effects on the acid–base balance.

#### 7.4.2. Effects of Bicarbonate-Rich Mineral Water on Bone Turnover

**Figure 6:**
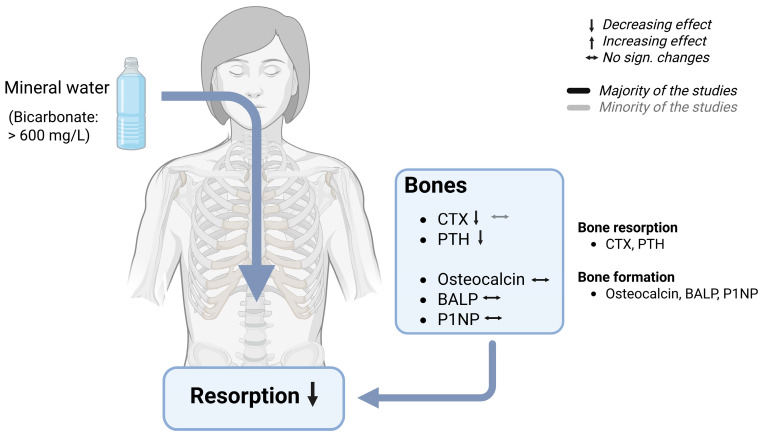
Effects of bicarbonate-rich mineral water on bone turnover. The main effects are reported with black arrows. Grey arrows indicate effects in the minority of the studies. CTX = C-terminal fragment of the type I collagen; BALP = bone specific alkaline phosphatase; P1NP = Procollagen type 1 N-terminal propeptide; PTH = parathyroid hormone.

Several randomized controlled trials have assessed the effects of bicarbonate-rich mineral water on bone metabolism, predominantly reporting favorable outcomes (Table 6, Figure 6). However, as already stated, the existing body of evidence suggests that these effects might result from the interplay between bicarbonate and calcium [53,56,117]. Studies examining the effects on bone metabolism have typically used mineral water rich in both calcium and bicarbonate, encompassing both calcium-deficient and calcium-sufficient participants. Moreover, these studies have evaluated the effects over a longer period of time, ranging from four days to eight weeks. In these studies, several markers reflecting both bone resorption and bone formation were assessed following a daily consumption of 1000–2000 mL of mineral water. Notably, none of the trials reported adverse effects, while several demonstrated beneficial effects [56,71,78]. Only one study failed to demonstrate positive effects [76].

However, it is imperative to acknowledge the limitations inherent in these studies. A single study was conducted in a larger cohort, encompassing 60 postmenopausal women [82]. Furthermore, it is to be assumed that none of the studies employed a blinding method, as no such information was reported in the papers.

Short-term studies ranging from 4 days to 4 weeks have shown that calcium- and bicarbonate-rich mineral water can reduce the biomarkers of bone resorption in humans [56,71,82]. While calcium-rich mineral water alone did not affect bone markers, mineral water rich in bicarbonate and calcium demonstrated beneficial effects on bone resorption, highlighting the beneficial influence of bicarbonate [56]. In this study, the bone resorption marker carboxyterminal type I collagen telopeptide (CTX) in blood samples decreased by 15% following the consumption of mineral water, showing significant group differences. In addition, urinary CTX showed a nonsignificant slight reduction, resulting in no significant group differences. However, in an earlier study, urinary CTX was shown to be reduced after bicarbonate-rich mineral water consumption with significantly lower levels after the bicarbonate-rich mineral phase and the control phases [71]. On the contrary, one study reported no significant differences in serum CTX after drinking a bicarbonate- and sodium-rich, but calcium-poor mineral water and a low mineralized mineral water [76]. On the other hand, there are indications that levels in the parathyroid hormone (PTH), a hormone responsible for bone resorption, can also be affected by mineral water consumption [57]. Three different randomized controlled trials have demonstrated a reduction in the parathyroid hormone (PTH), a hormone involved in bone demineralization and degradation of bone substance [43]. In postmenopausal women, ionized PTH levels in plasma decreased by 11% [82], while in young women, plasma PTH decreased by 16% [56] after the consumption of either 1000 or 1500 mL of bicarbonate and calcium-rich mineral water. However, one of these studies showed no significant group differences, indicating no different water effects [82]. On the contrary, the other study showed the superiority of bicarbonate-rich mineral water over a low mineralized mineral water [56]. In addition, in middle-aged men and women, a slightly higher intake of a bicarbonate- and calcium-rich mineral water (2000 mL/d) also led to a significant reduction of PTH in comparison to a mineral water poor in bicarbonate and calcium [71].

However, bone health is a constant interplay between bone resorption and bone formation. Hence, the effect on several bone-forming substances was also examined. In contrast to the biomarkers of bone resorption, bone-forming biomarkers, namely osteocalcin, bone-specific alkaline phosphatase (BALP), and procollagen type 1 N-terminal propeptide (P1NP), remained unchanged following the consumption of calcium- and bicarbonate-rich or bicarbonate- and sodium-rich mineral water [56,71,76,82].

### 7.5. Mechanism of Bone Protection by Bicarbonate-Rich Mineral Water

One possible mechanism underlying the bone-protective effects of bicarbonate-rich mineral water may be due to its pH-increasing properties. Evidence is derived from in vitro studies. In these studies, it has been shown that both osteoclasts, bone cells responsible for bone dissolution, and osteoblasts, bone cells responsible for bone formation, respond to changes in pH. Osteoclasts work best at a lower pH (around 7.0), and become progressively less active with increasing pH. Above pH 7.4, resorption is switched off. On the contrary, osteoblasts work best at a pH of 7.4, and osteoblast function is decreased by metabolic acidosis due to a decreased expression of extracellular matrix genes [118,119,120,121]. In brief, hydrogen ions generated from the digestion of foods rich in acid-forming precursors bind to osteoblasts and trigger the release of the receptor activator of the nuclear factor kappa-B ligand (RANK-L). This, in turn, promotes an increase in the number and activation of osteoclasts, thereby activating bone resorption [8]. This association could potentially be reversed or positively influenced by an alkaline diet or adequate consumption of bicarbonate-rich mineral water. Thus, the pH-increasing effects may modulate osteoclast activity and reduce bone resorption. Furthermore, the bone-protective effects may be partly mediated by the PTH-lowering properties of calcium- and bicarbonate-rich mineral water, thereby protecting against bone resorption.

Moreover, it has been postulated that decreasing the system pH increases the release of calcium from bones [122]. However, opponents of this acid–ash bone theory question the extent of this interrelationship. They argue that the higher urinary calcium, as it occurs under acidic diets, may also stem from increased intestinal calcium absorption, and not necessarily from the increased calcium loss that is caused by increased bone resorption [18,36].

#### 7.5.1. Effects of Bicarbonate-Rich Mineral Water on Bone Density and Fracture Risk

Despite the observed short-term effects on the markers of bone resorption, the long-term effects of consuming bicarbonate- and calcium-rich mineral water on bone density and fracture risk remain unclear [18,43]. There is a lack of observational and interventional studies examining this aspect specifically in the context of mineral water consumption over a longer period of time. Nevertheless, its bone-protective effect is conceivable, as indicated by a meta-analysis suggesting the potential benefits of alkaline supplements. This analysis revealed that interventions with alkaline supplements significantly increased bone mineral density at the femoral neck, lumbar spine, and total hip [36]. Similar results were observed in a longitudinal analysis that pooled data from two Mediterranean populations of middle-aged and older individuals. An analysis of the data revealed an increase in bone mass at different sites (total femur, femoral neck, femoral diaphysis, trochanter) associated with an acid-neutral diet (PRAL = 0) [123]. Nevertheless, the evidence of the relationship between dietary acid load and fracture risk remains inconclusive. The same longitudinal analysis reported a reduced risk of osteoporotic fractures with an acid-neutral diet. However, a low dietary PRAL score, which indicates an alkaline diet, was also associated with an increased risk of fractures [123]. By contrast, another meta-analysis found no association between fracture risk and adherence to an acid-producing diet. In addition, one study observed a weak association between NEAP and bone mineral density, while another study found no association between PRAL and bone mineral density [3].

#### 7.5.2. Bicarbonate-Rich Mineral Water and the Complexity with Other Nutrients Regarding Bone Health

In the context of mineral water and its potential effect on bone health, several components may contribute to positive effects. One such element is the calcium content, as mentioned above. In addition, the high potassium content of mineral water has been suggested to contribute to bone health [53]. However, research using low-potassium and high bicarbonate water has also demonstrated a successful reduction in bone resorption [82]. In addition, the potassium content of most water is generally considered to be insufficient to significantly affect bone metabolism [43].

Conversely, the relationship between fluoride-rich water and bone mineral density and fracture risk has also been studied in epidemiological studies. Some of the results suggest a potential positive association between fluoride intake and bone mineral density/fracture risk [124,125]. However, other studies could not confirm these associations [126,127,128]. In fact, there was even an increased fracture risk observed in postmenopausal women [125]. However, it is important to note that these findings specifically relate to fluoridated drinking water, not mineral water [43].

## 8. Summary Bicarbonate-Rich Mineral Water

Acid–base balance, a fundamental aspect of human health, can be influenced by the choice of consumed foods and beverages. A dietary pattern characterized by a high intake of fruits and vegetables has been shown to contribute to a balanced acid–base status by reducing the acid load contrast, while the frequent consumption of meat, cheese and cereals is associated with an increased dietary acid load. Given the propensity of the modern Western diet to induce excess acidity, there is a need to explore strategies aimed at mitigating this acidity. Consequently, considerable attention has been devoted in recent decades to evaluate the effects of bicarbonate-rich mineral water on the acid–base balance and related diseases.

Taken together, the findings from various scientific reports have indicated that mineral water with a bicarbonate level of more than 1300 milligrams per liter and a medium or low PRAL value exerts a beneficial and multifaceted effect on the urinary and blood parameters. One of the primary effects is the increase in the urine pH, caused by the enhancement of the body’s alkali reserves due to bicarbonate and mineral intake. This alkalization lowers renal NAE by reducing the excretion of TA and NH_4_^+^, while simultaneously increasing HCO_3_^−^ excretion. These changes in the urinary composition contribute to decreased urinary concentrations of stone-forming constituents, such as calcium and oxalates, with a more pronounced effect seen on oxalates. Conversely, the excretion of inhibitory substances, such as magnesium and citrate, are promoted. Moreover, it has been demonstrated that the alkalinization leads to a reduction in the bone resorption marker CTX, potentially by a reduction in osteoclast activation and a decrease in PTH levels. In terms of blood parameters, the consumption of bicarbonate-rich water stabilizes the blood pH while increasing the blood bicarbonate levels and the base excess, indicating a strengthened bicarbonate buffer system. This is achieved through bicarbonate absorption in the gastrointestinal tract, which enhances systemic buffering capacity and modulates pH, supporting general acid–base homeostasis.

From a clinical perspective, these changes lead to a lower risk of CaOx stone formation and promote kidney health due to an increase in the urine pH and favorable alterations in the urine composition. By preventing excessive crystallization and agglomeration of calcium and oxalates within the urinary tract, bicarbonate-rich mineral water is particularly beneficial for individuals with a history of CaOx urolithiasis. Additionally, by promoting an alkaline environment that favors osteoblastic activity and impedes osteoclastic activity, bicarbonate-rich mineral water can support bone health, although further research is required to fully assess its impact on bone density and fracture risk over time.

The studies included in this review exhibit several methodological limitations that must be considered when interpreting the overall findings. Most notably, the majority of the studies had a relatively small sample sizes, typically around 20 participants, which limits statistical power and generalizability. Only a few studies included more than 50 participants, reducing confidence in the robustness of the reported effects. Another important limitation concerns the study design quality. Randomization was not consistently applied across the studies, and information regarding blinding was often missing. Specifically, only five studies reported using a double-blind design, another five were single-blinded, five studies explicitly stated that no blinding was used, and, in eight studies, blinding procedures were not described at all. The frequent lack of blinding introduces a substantial risk of performance and detection bias. Furthermore, nearly half of the studies employed a parallel-group design rather than a crossover design (14 crossover vs. 9 parallel-group studies). While both designs have their merits, crossover trials generally offer increased statistical efficiency in within-subject comparisons, which is particularly valuable in studies with small sample sizes. The use of a crossover design may therefore represent a missed opportunity to enhance internal validity and reduce inter-individual variability. Collectively, these limitations highlight the need for more rigorously designed, adequately powered, and transparently reported trials in this field. Future studies should be conducted as randomized, double-blind, controlled studies in a crossover design. Additionally, a sufficient high sample size is imperative to enhance the statistical significance and the study’s generalizability, making the findings more representative of the broader population, and improving the external validity of the results. As the extant literature has predominantly focused on the effects of very high drinking volumes, it is imperative that future studies also examine the consequences of lower volumes. Finally, it is essential to record the diet using food frequency questionnaires or 3-day dietary protocols. However, the implementation of a controlled diet would be a superior approach.

In conclusion, bicarbonate-rich mineral water offers considerable benefits for acid–base regulation. In this context it appears to play a significant role in optimizing the urinary and blood parameters, offering protective effects against certain health conditions. Its incorporation into the diet as a strategy to counteract dietary-induced acidity, lowering kidney stone risk, and enhancing skeletal integrity holds promise. However, further methodologically improved studies are necessary to fully elucidate these benefits and to optimize recommendations for long-term health promotion.

## Figures and Tables

**Table 2:** Composition of selected bicarbonate-rich mineral water and calculation of the PRAL values. The mineral water brands are arranged according to the increasing PRAL value. The PRAL was calculated by the authors using the following equation [56]: PRAL (mEq/L) = 0.0146 × SO_4_^2−^ (mg/L) + 0.027 × Cl^−^ (mg/L) − 0.021 × K^+^ (mg/L) − 0.026 × Mg^2+^ (mg/L) − 0.0413 × Na^+^ (mg/L) − 0.013 × Ca^2+^ (mg/L).

Brand	Country	Na	K	Ca	Mg	Cl	SO_4_^2−^	P	HCO_3−_	PRAL
Rogaska Donat	Slovenia	1500	13	380	1030	59	2400	---	7700	−57.3
St-Yorre	France	1708	110	90	11	322	174	0	4368	−63.1
Borjomi	Georgia	1478	30	85	85	393	50		3965	−53.6
Adelheidquelle, Adelholzener	Germany	950	47.2	152	102	112	317	0	2999	−37.2
Vichy Celestins	France	1172	66	103	10	235	138	---	2989	−43.0
Heppinger Extra Medicinal Water	Germany	481	27.1	150	199	118	60	0.06	2495	−23.5
Jamnica	Croatia	921	32	115	34	252	109	---	2247	−32.7
Ardesy (Arvie)	France	650	130	170	92	387	31	---	2195	−23.3
Radenska	Slovenia	400	70	220	95	44	72	---	2000	−21.1
Rozana	France	493	52	301	160	649	230	---	1837	−8.6
Kryniczanka	Poland	43	5	436	68	19	8	---	1818	−8.7
Gerolsteiner	Germany	118	11	348	108	40	38	---	1816	−10.8
Apollinaris	Germany	470	30	90	120	130	100	0	1800	−19.4
Rhodius	Germany	137	33	143	151	22	37	---	1562	−11.0
Ferrarelle	Italy	50	50	392	22	20	4	---	1433	−8.2
Kalnicka	Croatia	650	8	62	23	350	0		1410	−19.0
Badoit	France	165	10	190	85	44	38	0	1300	−10.0
Rhäzünser	Switzerland	149	7	224	46	18	131	---	1120	−8.0
Verniere	France	110	40	180	173	14	140		1100	−9.8
Quezac	France	110	0	170	69	0	0	---	1100	−8.5
Sangemini	Italy	19.6	3.9	323	16.5	18.5	61	0	996	−4.1
Lete Acqua minerale	Italy	4.9	2.1	313	15.1	8.2	6.6	0	981	−4.4

**Table 3:** Effects of bicarbonate-rich mineral water on urine pH and NAE.

Author	Design Target Group	Intervention	Characteristics of Mineral Water/Treatment (Rich in) Bicarbonate/Day	Main Results	
				Time Effects * (Bicarbonate Group)	Group Differences Time × Group Interaction
Sub-chronic studies					
Schorr et al., 1996 [70]	Cross-over, randomized, double-blind 21 healthy older (60–72 years) subjects	NaCl reduction (<100 mmol/d) + 4 weeks (each) 3 different mineral water brands 1.5 L/d	Water A: HCO_3_, Na, Mg Water B: HCO_3_, Na, Cl Water C: low mineralized HCO_3_ Water A: 2975 mg/d Water B: 1318 mg/d Water C: 18 mg/d	**24-h urine**	
				**NAE**: (water A) ↔ (water B) *↑*	Week 4 **NAE**: n.s. group differences
Marangella et al., 1996 [71]	Cross-over, randomized 21 subjects with idiopathic calcium nephrolithiasis	1 month (each) 3 different mineral water brands Standardized diet 2 L/d	Water A: HCO_3_, Ca Water B: SO_4_ Water C: low mineralized HCO_3_ Water A: 3051 mg/d Water B: 610 mg/d Water C: 31 mg/d	**24-h urine**	
				**pH**: ↑ **NAE**: ↓	**pH**: water A > water B **NAE**: water A < water B and C
Caudarella et al., 1998 [72]	Cross-over 22 subjects with idiopathic calcium nephrolithiasis	20 days (each) 3 different mineral water brands Standardized diet 2 L/d	Water A: HCO_3_, Ca Water B: SO_4−_rich Water C: low mineralized HCO_3_ Water A: 2794 mg/d Water B: 610 mg/d Water C: 100 mg/d	**Fasting morning urine**	
				**pH**: ↔	**pH**: n.s. group differences
Keßler and Hesse, 1998, 2000 [73,81]	Cross-over, randomized 24 kidney-healthy younger (23–38 years) men	Run-In (standardized diet) + 2 days treatment (standardized diet) 4 weeks follow-up, same as cross-over week 2 (usual diet) Mineral water (”Staatl. Fachingen”) vs. supplement 2 L/d	Water: HCO_3_, Na, Mg Supplement: K citrate HCO_3_ Water 3430 mg/d K citrate: not reported	**24-h urine**	
				**Standardized diet** **pH**: ↑ **Usual diet** **pH**: ↑	Not reported
Coen et al., 2001 [74]	Parallel-group 21 healthy subjects	2 weeks 2 different mineral water brands Standardized diet 2 L/d	Water A: HCO_3_, Ca Water B: low mineralized HCO_3_ Water A: 2780 mg/d Water B: 121 mg/d	**Spot urine**	
				**pH**: ↔	**pH**: n.s. time × group interaction
Siener et al., 2004 [75]	Cross-over (first control, later mineral water) + single-arm 12 young healthy male subjects	2 weeks baseline (usual diet/beverages) 5 days cross-over (standardized diet); Mineral water vs. fruit tea (control) 4 weeks follow-up with mineral water (usual diet/beverages) 1.4 L/d	Water: HCO_3_, Ca, Mg, Na HCO_3_ Water: 4743 mg/d	**24-h urine**	
				**Standardized diet** **pH**: ↑ **NH_4_**: ↓ **Usual diet** **pH:** ↑ **NH_4_**: ↓	**Standardized diet** **pH**: water > control **NH_4_**: water < control ----
Roux et al., 2004 [82]	Cross-over, randomized 60 postmenopausal women	4 weeks (each) 2 different mineral water brands 1 L/d	Water A: HCO_3_, Ca Water B: Ca, Mg, SO_4_ HCO_3_ Water A: 2179 mg/d Water B: 292 mg/d	**2-h fasting urine**	
				**pH**: ↑ **TA- HCO_3_**: ↓ **NH_4_**: ↓	**pH**: water A > B **TA- HCO_3_:** water A < water B **NH_4_**: water A < water B
Schoppen et al., 2005 [76]	Cross-over 18 postmenopausal women	8 weeks (each) 2 different mineral water brands 1 L/d	Water A: HCO_3_, Na, Cl Water B: low mineralized HCO_3_ Water A: 2094 mg/d Water B: 71 mg/d	**24-h urine**	
				Not reported	**pH:** water A > water B
Karagülle et al., 2007 [77]	Cross-over, double-blind 34 subjects with multiepisodic CaOx stone formation	3 days (each) 2 different mineral water brands (water A: “Heppinger”, water B: “Bad Harzburger Urquell”) 1.5 L/d	Water A: HCO_3_, Mg, Na, Cl Water B: low mineralized HCO_3_ Water A: 4010 mg/d Water B: 149 mg/d	**24-h urine**	
				**pH**: ↑	**pH**: water A > water B
Wynn et al., 2009 [56]	Parallel-group, randomized 30 young (18–45 years) women	4 weeks 2 different mineral water brands (water A: “Adelbodner”; water B: “Kryniczanka”) Standardized diet 1.5 L/d	Water A: HCO_3_, Ca, Mg Water B: Ca, SO_4_ HCO_3_ Water A: 3258 mg/d Water B: 437 mg/d	**24-h urine**	
				**pH**: ↑ **HCO_3_**: ↑	**pH**: water A > water B **HCO_3_**: water A > water B
Perez-Granados et al., 2010 [78]	Cross-over (first water B, later water A), single-blind 18 young (>18–<40 years) hypercholesterolemic subjects	8 weeks (each) 2 different mineral water brands 1 L/d	Water A: HCO_3_, Na, Cl Water B: low mineralized HCO_3_ Water A: 2120 mg/d Water B: 104 mg/d	**24-h urine**	
				Not reported	**pH**: water A > water B
Brancaccio et al., 2012/12 [83,84]	Parallel-group 88 amateur athletes	7 days 2 different mineral water brands (water A “Aqua Lete”, water B) Repeated Wingate Tests (cycling) 1.5 L/d + 750 mL 1 h before exercise + 250 mL after exercise	Water A: HCO_3_, Ca Water B: low mineralized HCO_3_ (1.5 L) Water A: 1472 mg/d Water B: 5 mg/d	**Urine** (mixture of several time points)	
				Not reported During the day at the end of the intervention **pH**: ↑	**pH**: no changes over time with water B ^‡^
Toxqui and Vaquero, 2016 [85]	Cross-over, randomized, single-blind 64 moderately hypercholesterolemic men and women	8 weeks (each) 2 different mineral water brands 1 L/d	Water A: HCO_3_, Na, Cl Water B: low mineralized HCO_3_ Water A: 2050 mg/d Water B: 75 mg/d	**Fasting morning urine**	
				**pH**: ↑	**pH**: sign. time × group interaction
Wasserfurth et al., 2019 [13]	Parallel-group, randomized 129 healthy subjects	4 weeks 4 different mineral water brands 1.5–2 L/d	Water A: HCO_3_, Ca, Mg Water B: HCO_3_, Ca, Mg, Na Water C: HCO_3_, Mg, Na Water D: Ca, Mg, SO_4_ HCO_3_ (1.5 L) Water A: 2724 mg/d Water B: 3677 mg/d Water C: 2769 mg/d Water D: 605 mg/d	**24-h urine**	
				**pH**: ↔ (water A) (*p* = 0.068) ↑ (water B and C) **TA**: ↓ (water A-C) **HCO_3_**: ↑ (water A and B) ↔ (water C) **NH_4_**: ↓ (water A-C) **NAE**: ↓ (water A-C)	Week 4 **pH**: sign. group differences **TA**: n.s. group differences **HCO_3_**: sign. group differences **NH_4_**: n.s. group differences (*p* = 0.052) **NAE**: sign. group differences
				**Spontaneous urine**	
				**pH**: ↑ (water A and C)	**pH**: sign. group differences
Chycki et al., 2021 [48]	Cross-over (first table water, then HCO3 water), single-blind 8 elite judo athletes	3 weeks (each) Mineral water (water A) vs. table water (water B) Standardized meals Tests under hydrated conditions, treadmill to induce hypohydration, anaerobic Wingate tests under dehydrated conditions, later rehydration Amount individualized, approx. 3.2–3.4 L/d	Water A: HCO_3_, Na, Cl Water B: low mineralized HCO_3_ (3.3 L) Water A: 13,207 mg/d Water B: 12 mg/d	**24-h urine**	
				Not reported	Post-supplementation time point **Hydrated + dehydrated condition** **pH**: n.s. group differences (trend: water A > water B)
Lu et al., 2022 [86]	Parallel-group, randomized 58 subjects with Ca stones	12 weeks Mineral water (water A: “Ardesy”) vs. tap water (water B) 1.25 L/d	Water A: HCO_3_, Ca, Mg, Na, Cl Water B: low mineralized HCO_3_ Water A: 2744 mg/d Water B: not reported	**24-h urine**	
				(no *p*-values reported) **pH**: ↑ (trend) ^†^	Week 12 ^§^ **pH**: n.s. group differences (*p* = 0.071)
Chiron et al., 2024 [59]	Parallel group (diet) Cross-over (water, within a diet group), randomized, double-blind 24 recreationally active men	7 days 2 different mineral water brands (water A: “St-Yorre”, water B) Dietary restrictions (alkalizing diet vs. acidifying diet) 1-min supra-maximal rowing Wingate Test 1.5–2 L/d	Water A: HCO_3_, Na, Cl Water B: low mineralized HCO_3_ (2 L) Water A: 8736 mg/d Water B: 612 mg/d	**24-h urine**	
				Not reported	Post-supplementation time point **Water effects (whole group)** ^#^ **pH**: water A > water B **Water effects (alkalizing diet)** **pH**: water A > water B **Water effects (acidifying diet)** **pH**: water A > water B
Mansouri et al., 2023, 2024 [58,87]	Parallel-group, randomized 94 healthy subjects	4 weeks 2 different mineral water brands 1.5–2 L/d	Water A: HCO_3_, Na, Cl Water B: low mineralized HCO_3_ (1.5 L) Water A: 6552 mg/d Water B: 342 mg/d	**24-h urine**	
				**pH**: ↑ **TA**: ↓ **NH_4_**: ↓ **HCO_3_**: ↑ **NAE**: ↓	**pH**: sign. time × water interaction (water A ↑, water B ↔) **TA**: sign. time × water interaction (water A < water B) **NH_4_**: sign. time × water interaction (water A < water B) **HCO_3_**: sign. time × water interaction (water A ↑; water B ↓) **NAE**: sign. time × water interaction (water A < water B)
Chiron et al., 2024 [79]	Parallel-group, randomized, double-blind 22 highly trained athletes	6 days 2 different mineral water brands (water A: “St-Yorre”, water B) Dietary restrictions (alkalizing diet) Last 3 days: 400 m race + handgrip strength + squat jumps (each day) 4 × 500 mL /d	Water A: HCO_3_, Na, Cl Water B: low mineralized HCO_3_ Water A: 8736 mg/d Water B: 612 mg/d	**24-h urine**	
				**pH**: ↑	**pH**: water A > water B
Acute studies					
Schoppen et al., 2008 [80]	Cross-over, randomized 18 postmenopausal women	--- 3 different mineral water brands 500 mL	Water A: HCO_3_, Na (higher than water B), Cl Water B: HCO_3_, Na, Cl Water C: low mineralized HCO_3_ Water A: 1047 mg Water B: 1007 mg Water C: 36 mg	**Postprandial urine**	
				Not reported	**pH**: n.s. group differences

Minerals: HCO_3_ = bicarbonate; Ca = calcium; Mg = magnesium; Na = sodium; Cl = chloride; SO_4_ = sulfate. Urinary parameters: Ca = calcium; TA = titratable acid; NH_4_ = ammonium; HCO_3_ = bicarbonate; NAE = net acid excretion. Notes: ↑ = significant increase (*p* < 0.05); ↔ = no significant change (*p* > 0.05); ↓ = significant decrease (*p* < 0.05); * between begin and end of each intervention period; ^†^ categorization of the authors; ^‡^ no *p*-values reported for group differences; ^§^ time effects extracted from tables reporting changes between baseline and different weeks (no *p*-values reported) + text, group differences are calculated between changes at one time point; ^#^ adjusted for diet group.

**Table 4:** Effects of bicarbonate-rich mineral water on stone risk.

Author	Design Target Group	Intervention	Characteristics of Mineral Water/ Treatment Bicarbonate/Day	Main Results	
				Time Effects * (Bicarbonate Group)	Group Differences Time × Water Interaction
Sub-chronic studies					
Luft et al., 1990 [95]	Cross-over, randomized, single-blind 10 subjects (hypertensive + normotensive)	4 days Run-In + 7 days (each) Mineral water (“Staatl. Fachingen”) vs. control solution (NaCl) Standardized diet (low sodium, low calcium) 3 L/d	Water: HCO_3_, Na, Mg Control solution: Na, Cl, Mg HCO_3_ Water A: 6046 mg/d Water B: 0 mg/d	**Urine** (not specified)	
				Not reported	Ca: sign. group differences water < NaCl normotensives < hypertensives blacks < whites
Schorr et al., 1996 [70]	Cross-over, randomized, double-blind 21 healthy older (60–72 years) subjects	NaCl reduction (<100 mmol/d) + 4 weeks (each) 3 different mineral water brands 1.5 L/d	Water A: HCO_3_, Na, Mg Water B: HCO_3_, Na, Cl Water C: low mineralized HCO_3_ Water A: 2975 mg/d Water B: 1318 mg/d Water C: 18 mg/d	**24-h urine**	
				**NAE:** (water A) ↔ (water B) ↑ **Ca:** (water A) ↓ (water B) ↔	Week 4 **NAE:** (water A): n.s. group differences (water B): n.s. group differences **Ca:** (water A): n.s. group differences (water B): n.s. group differences
Marangella et al., 1996 [71]	Cross-over, randomized 21 subjects with idiopathic calcium nephrolithiasis	1 month (each) 3 different mineral water brands Standardized diet 2 L/d	Water A: HCO_3_, Ca Water B: SO_4_ Water C: low mineralized HCO_3_ Water A: 3051 mg/d Water B: 610 mg/d Water C: 31 mg/d	**24-h urine**	
				**pH**: ↑ **Ca**: ↑ **Oxalate**: ↓ **Citrate**: ↑ **Mg**: ↑ **RS CaOx**: ↓	**pH**: water A > water B **Ca**: water A > water B **Oxalate**: water A < water B and C **Citrate**: water A > water B and C **Mg**: water A > water B and C **RS CaOx**: n.s. group differences
Caudarella et al., 1998 [72]	Cross-over 22 subjects with idiopathic calcium nephrolithiasis	20 days (each) 3 different mineral water brands Standardized diet 2 L/d	Water A: HCO_3_, Ca Water B: SO_4_-rich Water C: low mineralized HCO_3_ Water A: 2794 mg/d Water B: 610 mg/d Water C: 100 mg/d	**Fasting morning urine**	
				**pH**: ↔ **RS CaOx**: ↔	**pH**: n.s. group differences **RS CaOx**: n.s. group differences
				**24-h urine**	
				**Ca**: ↔ (tendency ↑) ^†^ **Oxalate**: ↓ **Mg**: ↑	**Ca**: n.s. group differences **Oxalate**: n.s. group differences **Mg**: water A > water B
				**Urine** (not specified)	
				**Citrate**: ↑	**Citrate**: n.s. group differences
Keßler and Hesse, 1998, 2000 [73,81]	Cross-over, randomized 24 kidney-healthy younger (23–38 years) men	Run-In (standardized diet) + 2 days treatment (standardized diet) 4 weeks follow-up, same as cross-over week 2 (usual diet) Mineral water (”Staatl. Fachingen”) vs. supplement 2 L/d	Water: HCO_3_, Na, Mg Supplement: K Citrate HCO_3_ Water 3430 mg/d K Citrate: not reported	**24-h urine**	
				**Standardized diet** **pH**: ↑ **Ca**: ↔ (tendency ↓) ^†^ **Oxalate**: ↓ **Citrate**: ↑ **Mg**: ↔ **RS CaOx**: ↓ **RS UA**: ↓ **Usual diet** **pH**: ↑ **Ca**: ↔ (tendency ↓) ^†^ **Oxalate**: ↔ (tendency ↓) ^†^ **Citrate**: ↑ **Mg**: ↔ (tendency ↑) ^†^ **RS CaOx**: ↓ **RS UA**: ↓	Day 5 **Standardized diet** n.s. group differences Week 4 **Usual diet** n.s. group differences
Coen et al., 2001 [74]	Parallel-group 21 healthy subjects	2 weeks 2 different mineral water brands Standardized diet 2 L/d	Water A: HCO_3_, Ca Water B: low mineralized HCO_3_ Water A: 2780 mg/d Water B: 121 mg/d	**Spot urine**	
				**pH**: ↔ **Ca**: ↔ **RS CaOx (Tiselius Index)**: ↔	**pH**: n.s. time × group interaction **Ca**: sign. time × group interaction (water A > water B) **RS CaOx (Tiselius Index)**: n.s. time × group interaction
				**24-h urine**	
				**Oxalate**: ↔ (tendency: ↑ *p* = 0.068) ^†^ **Citrate**: ↑ **Mg**: ↔ (tendency: ↑ *p* = 0.058) ^†^	**Oxalate**: n.s. time × group interaction **Citrate**: n.s. time × group interaction **Mg**: n.s. time × group interaction
Siener et al., 2004 [75]	Cross-over (first control, later mineral water) + single-arm 12 young healthy male subjects	2 weeks baseline (usual diet/beverages) 5 days cross-over (standardized diet); Mineral water vs. fruit tea (control) 4 weeks follow-up with mineral water (usual diet/beverages) 1.4 L/d	Water: HCO_3_, Ca, Mg, Na HCO_3_ Water: 4743 mg/d	**24-h urine**	
				**Standardized diet** **pH**: ↑ **Ca**: ↑ **Oxalate**: ↔ **Citrate**: ↑ **Mg**: ↑ **RS CaOx**: ↔ **Usual diet** **pH**: ↑ **Ca**: ↔ **Oxalate**: ↔ **Citrate**: ↑ **Mg**: ↑ **RS CaOx**: ↓	**Standardized diet** **pH**: water A > control **Ca**: water A > control **Oxalate:** n.s. group differences **Citrate**: water A > control **Mg**: water A > control **RS CaOx**: n.s. group differences ----
Schoppen et al., 2005 [76]	Cross-over 18 postmenopausal women	8 weeks (each) 2 different mineral water brands 1 L/d	Water A: HCO_3_, Na, Cl Water B: low mineralized HCO_3_ Water A: 2094 mg/d Water B: 71 mg/d	**24-h urine**	
				Not reported	pH: water A > water B Ca: water A < water B
Karagülle et al., 2007 [77]	Cross-over, double-blind 34 subjects with multiepisodic CaOx stone formation	3 days (each) 2 different mineral water brands (water A: “Heppinger”, water B: “Bad Harzburger Urquell”) 1.5 L/d	Water A: HCO_3_, Mg, Na, Cl Water B: low mineralized HCO_3_ Water A: 4010 mg/d Water B: 149 mg/d	**24-h urine**	
				**pH**: ↑ **Ca:** ↑ **Oxalate**: ↑ **Citrate**: ↑ **Mg**: ↑ **RS CaOx**: ↓	**pH**: water A > water B **Ca**: n.s. group differences **Oxalate**: n.s. group differences **Citrate**: water A > water B **Mg**: water A > water B **RS CaOx**: n.s. group differences
Wynn et al., 2009 [56]	Parallel-group, randomized 30 young (18–45 years) women	4 weeks 2 different mineral water brands (water A: “Adelbodner”, water B: “Kryniczanka”) Standardized diet 1.5 L/d	Water A: HCO_3_, Ca, Mg Water B: Ca, SO_4_ HCO_3_ Water A: 3258 mg/d Water B: 437 mg/d	**24-h urine**	
				**pH**: ↑ **Ca**: ↑	**pH**: water A > water B **Ca**: water A < water B
Toxqui and Vaquero, 2016 [85]	Cross-over, randomized, single-blind 64 moderately hypercholesteremic men and women	8 weeks (each) 2 different mineral water brands 1 L/d	Water A: HCO_3_, Na, Cl Water B: low mineralized HCO_3_ Water A: 2050 mg/d Water B: 75 mg/d	**Fasting morning urine**	
				**pH**: ↑ **Ca/creatinine**: ↓	**pH**: sign. time × group interaction **Ca/creatinine**: sign. time × group interaction
Wasserfurth et al., 2019 [13]	Parallel-group, randomized 129 healthy subjects	4 weeks 4 different mineral water brands 1.5–2 L/d	Water A: HCO_3_, Ca, Mg Water B: HCO_3_, Ca, Mg, Na Water C: HCO_3_, Mg, Na Water D: Ca, Mg, SO_4_ HCO_3_ (1.5 L) Water A: 2724 mg/d Water B: 3677 mg/d Water C: 2769 mg/d Water D: 605 mg/d	**24-h urine**	
				**pH**: ↔ (water A) (*p* = 0.068) ↑ (water B and C) **Ca**: ↑ (water A, B and D) ↔ (water C)	**pH**: sign. group differences **Ca**: sign group differences
Lu et al., 2022 [86]	Parallel-group, randomized 58 subjects with Ca stones	12 weeks Mineral water (water A: “Ardesy”) vs. tap water (water B) 1.25 L/d	Water A: HCO_3_, Ca, Mg, Na, Cl Water B: low mineralized HCO_3_ Water A: 2744 mg/d Water B: not reported	**24-h urine**	
				**pH**: ↑ (trend) ^†^ **Ca**: ↑ **Oxalate**: ↓ **Citrate**: ↑ (trend) ^†^ **Mg**: ↑ (trend) ^†^ **RS CaOx (Tiselius Index)**: ↑ (No *p*-values reported)	Week 12 ^‡^ **pH**: n.s. group differences (*p* = 0.071) **Ca**: n.s. group differences **Oxalate**: n.s. group differences **Citrate**: n.s. group differences (*p* = 0.084) ^†^ **Mg**: water A > water B **RS CaOx (Tiselius Index)**: n.s. group differences (*p* = 0.060)
Mansouri et al., 2023, 2024 [58,87]	Parallel-group, randomized 94 healthy subjects	4 weeks 2 different mineral water brands 1.5–2 L/d	Water A: HCO_3_, Na, Cl Water B: low mineralized HCO_3_ (1.5 L) Water A: 6552 mg/d Water B: 342 mg/d	**24-h urine**	
				**pH**: ↑ **Ca**: ↓	**pH**: sign. time × water interaction (water A ↑, water B ↔) **Ca**: n.s. time × water interaction (*p* = 0.060)
Acute studies					
Schoppen et al., 2008 [80]	Cross-over, randomized 18 postmenopausal women	--- 3 different mineral water brands 500 mL	Water A: HCO_3_, Na (higher than water B), Cl Water B: HCO_3_, Na, Cl Water C: low mineralized HCO_3_ Water A: 1047 mg Water B: 1007 mg Water C: 36 mg	**Postprandial urine**	
				Not reported	**pH**: n.s. group differences **Ca**: n.s. group differences

Minerals: HCO_3_ = bicarbonate; Ca = calcium; Mg = magnesium; Na = sodium; Cl = chloride; SO_4_ = sulfate. Urinary parameters: Ca = calcium; Mg = magnesium; RS CaOx = calcium oxalate supersaturation. Notes: ↑ = significant increase (*p* < 0.05); ↔ = no significant change (*p* > 0.05); ↓ = significant decrease (*p* < 0.05); * between begin and end of each intervention period; ^†^ categorization of the authors; ^‡^ time effects extracted from tables reporting changes between baseline and different weeks (no *p*-values reported) + text, group differences are calculated between changes at one time point.

**Table 5:** Effects of bicarbonate-rich mineral water on blood gas parameters.

Author	Design Target Group	Intervention	Characteristics of Mineral Water/Treatment Bicarbonate/day	Main Results	
				Time Effects * (Bicarbonate Group)	Group Differences Time × Water Interaction **
Sub-chronic studies					
Wasserfurth et al., 2019 [13]	Parallel-group, randomized 129 healthy subjects	4 weeks 4 different mineral water brands 1.5–2 L/d	Water A: HCO_3_, Ca, Mg Water B: HCO_3_, Ca, Mg, Na Water C: HCO_3_, Mg, Na Water D: Ca, Mg, SO_4_ HCO_3_ (1.5 L) Water A: 2724 mg/d Water B: 3677 mg/d Water C: 2769 mg/d Water D: 605 mg/d	**12-h fasting blood** (venous)	
				**pH**: ↔ (water B and C) ↓ (water A) **HCO_3_**: ↑ (water C) ↔ (water A and B) (water B *p* = 0.057) **BE**: ↑ (water C) ↔ (water A and B)	**pH**: sign. group differences **HCO_3_**: n.s. group differences **BE**: n.s. group differences
Chycki et al., 2021 [48]	Cross-over (first table water, later HCO_3_ water), single-blind 8 elite judo athletes	21 days (each) Mineral water (water A) vs. table water (water B) Standardized diet Anaerobic Wingate tests (high intensity) under hydrated + dehydrated conditions, treadmill to induce hypohydration, later rehydration Amount individualized, approx. 3.2–3.4 L/d	Water A: HCO_3_, Na, Cl Water B: low mineralized HCO_3_ (3.3 L) Water A: 13,207 mg/d Water B: 12 mg/d	**Blood** (capillary: fingertip)	
				Not reported During post-supplementation time point (no *p*-values reported) **pH** (resting): *↓* **HCO_3_** (resting): *↓*	Post-supplementation time point **Hydrated and dehydrated condition** **pH** (resting): n.s. group differences **HCO_3_** (resting): water A > water B
Hagele et al., 2023 [107]	Parallel-group, randomized, double-blind 39 recreationally active men and women	7 days Mineral water (water A: “Borjomi”) vs. spring water (water B) Same diet before each visit Anaerobic cycling 10 mL/kg, 40–60 min prior to exercise tests	Water A: HCO_3_, Na, Cl Water B: low mineralized HCO_3_ Water A: approx. 3000 mg/d Water B: not reported	**8-h fasting blood** (venous)	
				Not reported During post-supplementation time point **pH**: ↓ **HCO_3_**: ↓ **BE**: ↓	**pH**: n.s. time × group interaction (water A > water B at immediate post + 10 min post exercise) **HCO_3_**: n.s. time × group interaction **BE**: n.s. time × group interaction
Chiron et al., 2024 [59]	Parallel-group (diet) Cross-over (water, within a diet group), randomized, double-blind 24 recreationally active men	7 days 2 different mineral water brands (water A: “St-Yorre”, water B) Dietary restrictions (alkalizing diet vs. acidifying diet) 1-min supra-maximal rowing Wingate Test 1.5–2 L/d	Water A: HCO_3_, Na, Cl Water B: low mineralized HCO_3_ (2 L) Water A: 8736 mg/d Water B: 612 mg/d	**Blood** (capillary: earlobe)	
				Not reported During post-supplementation time point (no *p*-values reported) **Water effect** **pH**: ↓ **HCO_3_**: ↓ **Water effect (alkalizing diet)** Not reported **Water effects (acidifying diet)** Not reported	**Water effects (whole group)** ^†^ **pH**: water A > water B (immediately after + 3 min + 5 min post exercise) **HCO_3_**: n.s. group difference **Water effects (alkalizing diet)** **pH**: water A > water B (immediately after + 5 min post exercise) **HCO_3_**: water A > water B (warm-up + immediately post exercise) **HCO_3_ (peak)**: water A > water B **Water effects (acidifying diet)** **pH**: n.s. group differences **HCO_3_**: n.s. group differences
Mansouri et al., 2024 [58]	Parallel-group, randomized 94 healthy subjects	4 weeks 2 different mineral water brands 1.5–2 L/d	Water A: HCO_3_, Na, Cl Water B: low mineralized HCO_3_ (1.5 L) Water A: 6552 mg/d Water B: 342 mg/d	**12-h fasting blood** (venous)	
				**pH**: ↔ **HCO_3_**: ↑ **BE**: ↑	**pH**: n.s. time × group interaction **HCO_3_**: sign. time × group interaction (water A ↑, water B ↔) **BE**: sign. time × group interaction (water A ↑, water B ↔)
Chiron et al., 2024 [79]	Parallel-group, randomized, double-blind 22 highly trained athletes	6 days 2 different mineral water brands (water A: “St-Yorre”, water B) Dietary restrictions (alkalizing diet) Last 3 days: 400 m run + handgrip strength + squat jumps (each day) 4 × 500 mL/d	Water A: HCO_3_, Na, Cl Water B: low mineralized HCO_3_ Water A: 8736 mg/d Water B: 612 mg/d	**Blood** (capillary: fingertip)	
				Not reported During post-supplementation time point (no *p*-values reported) **pH**: ↓ **HCO_3_**: ↓ **BE:** ↓	**pH**: water A > water B (pre 400 m run) **HCO_3_**: water A > water B (HCO_3_ max) **BE:** water A > water B (1 h post 400 m run)
Acute Studies					
Richard et al., 2000 [108]	Cross-over, randomized, single-blind 12 regularly trained athletes	--- 3 different mineral water brands (water A: “St-Yorre”, water B, water C) Standardized meal Anaerobic cycling + isokinetic endurance test (after recovery) 3 L (1.5 l before exercise + 0.5 L during exercise + 1 L during recovery)	Water A: HCO_3_, Na Water B: Na water C: low mineralized HCO_3_ Water A: 13,104 mg/d Water B: not reported Water C: not reported	**Blood** (capillary)	
				(no *p*-values reported) **pH**: ↑ (pre—post cycling) ↓ (until end of isokinetic test) **HCO_3_**: ↓ (pre- post cycling) ↑ (recovery) ↓ (slightly, until end of isokinetic test)	**pH**: water A > water C (immediately after exercise + after isokinetic test) **HCO_3_**: water A > water C

Minerals: HCO_3_ = bicarbonate; Ca = calcium; Mg = magnesium; Na = sodium; Cl = chloride; SO_4_ = sulfate; Blood parameters: BE = base excess; HCO_3_ = bicarbonate. Notes: ↑ = significant increase (*p* < 0.05); ↔ = no significant change (*p* > 0.05); ↓ = significant decrease (*p* < 0.05); * between begin and end of each intervention period; ** at end of each intervention period; ^†^ adjusted for diet group.

**Table 6:** Effects of bicarbonate-rich mineral water on bone turnover.

Author	Design Target Group	Intervention	Characteristics of Mineral Water/Treatment Bicarbonate/Day	Main Results	
				Time Effects * (Bicarbonate Group)	Group Differences Time × Water Interaction
Sub-chronic studies					
Marangella et al., 1996 [71]	Cross-over, randomized 21 subjects with idiopathic calcium nephrolithiasis	1 month (each) 3 different mineral water brands Standardized diet 2 L/d	Water A: HCO_3_, Ca Water B: SO_4_ Water C: low mineralized HCO_3_ Water A: 3051 mg/d Water B: 610 mg/d Water C: 31 mg/d	**Fasting urine**	
				Not reported	**Hydroxyproline**: water A < water C **Cross-linked N-telopeptide type I**: water A < water B and C
				**Blood** (not specified)	
				Not reported	**PTH**: water A and B < water C **Osteocalcin**: n.s. group differences
Roux et al., 2004 [82]	Cross-over, randomized 60 postmenopausal women	4 weeks (each) 2 different mineral water brands 1 L/d	Water A: HCO_3_, Ca Water B: Ca, Mg, SO_4_ HCO_3_ Water A: 2179 mg/d Water B: 292 mg/d	**2-h fasting urine**	
				**pH**: ↑ **CTX/Cr**: ↓ **Pyr/Cr**: ↓	**pH:** water A > water B **CTX/Cr**: n.s. group differences **Pyr/Cr**: n.s. group differences
				**Fasting blood**	
				**iCa** (serum): ↑ **total Ca** (serum): ↔ **P** (serum): ↔ **iPTH** (plasma): ↓ **Osteocalcin** (serum): ↔ **BALP** (serum): ↔	No sign. group differences
Schoppen et al., 2005 [76]	Cross-over 18 postmenopausal women	8 weeks (each) 2 different mineral water brands 1 L/d	Water A: HCO_3_, Na, Cl Water B: low mineralized HCO_3_ Water A: 2094 mg/d Water B: 71 mg/d	**24-h urine**	
				Not reported	**pH**: water A > water B **Ca**: water A < water B
				**12-h fasting blood**	
				Not reported	**CTX** (serum): n.s. group differences **P1NP** (serum): n.s. group differences
Wynn et al., 2009 [56]	Parallel-group, randomized 30 young (18–45 years) women	4 weeks 2 different mineral water brands (water A: “Adelbodner”, water B: “Kryniczanka”) Standardized diet 1.5 L/d	Water A: HCO_3_, Ca, Mg Water B: Ca, SO_4_ HCO_3_ Water A: 3258 mg/d Water B: 437 mg/d	**24-h urine**	
				**pH**: ↑ **Ca**: ↑ **CTX**: ↔ (slightly ↓) ^†^	**pH**: water A > water B **Ca**: water A < water B **CTX**: n.s. group differences
				**Blood** (not specified)	
				**iCa**: ↔ **total Ca**: ↔ **P**: ↔ **CTX** (serum): ↓ **PTH** (plasma): ↓ **BALP**: ↔	**iCa**: n.s. group differences **total Ca**: n.s. group differences **P**: n.s. group differences **CTX** (serum): changes water A > water B **PTH** (plasma): changes water A > water B **BALP**: n.s. group differences

Minerals: HCO_3_ = bicarbonate; Ca = calcium; Mg = magnesium; Na = sodium; Cl = chloride; SO_4_ = sulfate. Urinary parameters: Ca = calcium; Cr = creatinine; CTX = C-terminal fragment of the type I collagen; NTX = cross-linked N-telopeptide type I; Pyr = pyridinoline. Blood parameters: BALP = bone specific alkaline phosphatase; CTX = C-terminal fragment of the type I collagen; iCa = ionized calcium; P = phosphate; P1NP = procollagen type 1 N-terminal propeptide; PTH = parathyroid hormone. Notes: ↑ = significant increase (*p* < 0.05); ↔ = no significant change (*p* > 0.05); ↓ = significant decrease (*p* < 0.05); * between begin and end of each intervention period; ^†^ categorization of the authors (no *p*-value reported).

## Abbreviations

PRAL	Potential Renal Acid Load
NEAP	Net Endogenous Acid Production
NAE	Net Acid Excretion
OA	Organic acids
TA	Titrable acids
NH_4_^+^	Ammonium
HCO_3_^-^	Bicarbonate
Ca	Calcium
Mg	Magnesium
Na	Sodium
K	Potassium
Cl	Chloride
SO_4_	Sulfate
P	Phosphorus
BE	Base Excess
CaOx	Calcium oxalate
CaP	Calcium phosphate
RS	Relative supersaturation
UA	Uric acid
Cr	Creatinine
CTX	C-terminal fragment of the type I collagen (CTX)
iCa	Ionized calcium
P1NP	Procollagen type 1 N-terminal propeptide
PTH	Parathyroid hormone
